# Supplementary material for: Optimizing multi-user indoor sound communications with acoustic reconfigurable metasurfaces
Source: Nat Commun. 2024 Feb 10;15:1270. doi: 10.1038/s41467-024-45435-4 (PMC10858938; doi:10.1038/s41467-024-45435-4)
Supplement: Supplementary file 1 — Supplementary Information [file 41467_2024_45435_MOESM1_ESM.pdf]

SUPPLEMENTARY INFORMATION

Optimizing multi-user indoor sound communications with acoustic reconfigurable metasurfaces

Hongkuan Zhang[1,4], Qiyuan Wang[1,4,5], Mathias Fink[2], Guancong Ma[1,3]

[1]Department of Physics, Hong Kong Baptist University, Kowloon Tong, Hong Kong

[2]Institut Langevin, ESPCI Paris, Université PSL, CNRS, Paris 75005, France

[3]Shenzhen Institute for Research and Continuing Education, Hong Kong Baptist University, Shenzhen 518000, China

[4]Contributed equally to this work.

[5]Present address: Graduate School of Engineering, The University of Tokyo, Japan

Contents

Supplementary Notes

1. On the reciprocity of channel matrices
2. Characterizing the sound field in the laboratory
3. Maximal channel capacity
4. The roles of the effective rank R_{eff} and parameter w_1 in $\mathcal{G}_1(\mathbf{H})$
5. Ensemble averages of R_{eff} and w_1
6. Measurement of R_{eff} and w_1 in the frequency range of 500-4000 Hz
7. Objective functions for optimizing 6×2 and 4×2 channel matrices
8. OCI over a continuous band of frequency
9. The optimization algorithm

Supplementary References

Supplementary Figures

Supplementary Notes

1. On the reciprocity of channel matrices

Here, we discuss the issue of reciprocity of channel matrices. The sound field in the room is reciprocal. However, in the channel matrix between finite number sources and receivers, reciprocity is not always present.

To begin, we establish that the room as an acoustic cavity is reciprocal. Sound propagation inside the cavity is governed by acoustic wave equation that is a quadratic differential equation in both space and time. Therefore, both spatial reciprocity and time-reversal symmetry hold. The inevitable presence of dissipation does slightly break time-reversal symmetry but not to the degree that it breaks reciprocity. This is evident by the successful time-reversal experiments performed in similar rooms[1,2].

Consider a channel matrix, denoted as $\mathbf{H}$ with h_{ij} being the entries, that connects sources ($\mathbf{S}$) and receivers ($\mathbf{R}$). When the positions of the sources and receivers are exchanged, they are connected by a new channel matrix $\mathbf{H}'$ with h'_{ij} being entries. It is straightforward to see that $\mathbf{H}' = \mathbf{H}^{\mathrm{T}}$. The reciprocity condition of channel matrices requires $\mathbf{H} = \mathbf{H}'$, which means $\mathbf{H}$ has transpose invariant, i.e., $\mathbf{H} = \mathbf{H}^{\mathrm{T}}$ (Supplementary Fig. 1a). In general, this condition is not satisfied. For instance, in a 2×2 channel matrix, there is no guarantee that $h_{12} = h'_{12}$ and $h_{21} = h'_{21}$. However, once channel isolation is attained, the channel matrix becomes a near-diagonal matrix with $h_{12} \cong h'_{12} \cong 0$, and $h_{21} \cong h'_{21} \cong 0$, so the matrix is nearly symmetric. Apparently, the reciprocity condition is largely satisfied (Supplementary Fig. 1b, left). The same clearly also holds for near anti-diagonal channel matrix (Supplementary Fig. 1b, right). Since channel isolation is same for communication in both directions, consequently, there is no need for re-optimization when exchanging the positions of the sound sources and the receivers. Such a property is desirable for real-life acoustic communications where the roles of source and receivers are often switching.

However, in scenarios where the numbers of sources and receivers are unequal (Supplementary Fig. 1c), there is no reciprocity in the channel matrix, because apparently $\mathbf{H} \neq \mathbf{H}^{\mathrm{T}}$ for rectangular matrices. Hence, re-optimization of the room configuration is in principle required when the positions of sources and receivers are exchanged.

2. Characterizing the sound field in the laboratory

The acoustic characteristics of the experimental environment are detailed in the Methods section. In this supplementary note, we provide additional information regarding the spatial uniformity and statistical characteristics of the sound field within the laboratory through experimental measurements.

First, we verify the homogeneity of the sound intensity in the room, which is an important criterion for acoustic reverberation. We have conducted measurements of the sound fields in 8 different planes (1.5-by-1.4 m^2) in the room. These measurements involved performing two-

dimensional raster scans across the frequency range from $f_1 = 250\ \mathrm{Hz}$ to $f_2 = 8000\ \mathrm{Hz}$. The spectral average of sound pressure levels (SPL), defined as

$$\mathrm{SPL} = 10\log_{10}\left[\frac{1}{f_2 - f_1}\int_{f_1}^{f_2} |p(f)|^2 \mathrm{d}f\right], \quad \text{(S1)}$$

is presented in Supplementary Fig. 2. Here, $p(f)$ refers to the sound pressure at frequency f. It is seen that the SPL is rather flat in space with some random undulations: the spatially-averaged SPL, denoted $\overline{\mathrm{SPL}}$, and the standard deviation $\sigma(\mathrm{SPL})$ for the 1000-2000 Hz range are -28.96 dB and 0.655 dB, respectively. For the 250-8000 Hz range, the corresponding values are -25.86 dB and 0.5615 dB, respectively. It has been accepted that an acoustic field in a qualified reverberation room exhibits adequate diffuseness if the standard deviations remain under 1.5 dB[3].

To further analyze the statistical wave characteristics, we have experimentally obtained the acoustic field distributions by performing several two-dimensional raster scans at different positions in the laboratory. The measured frequency is 1300 Hz. Four sets of results (1.0-by-1.0 m^2) are shown in Supplementary Fig. 3. From the results, we obtain that the distributions of the real and imaginary parts of the sound pressure, denoted by $\mathrm{Re}(p)$ and $\mathrm{Im}(p)$, follow a Gaussian distribution, i.e., $\rho_G(x) = \frac{1}{\sqrt{2\pi}}\mathrm{e}^{-\frac{1}{2}x^2}$; and the acoustic magnitudes, denoted by $|p|$, follow a Rayleigh distribution, i.e., $\rho_R(x) = x\mathrm{e}^{-\frac{1}{2}x^2}$, as shown in Supplementary Fig. 4(a, b, c). We have further computed the spatial correlation of the fields, as shown in Supplementary Fig. 4d. The correlation among real parts follows a *sinc* function with an FWHM of $\sim 0.3\,\lambda$, and the correlation between the real and imaginary parts is negligible. These properties suggest that the field is a circularly symmetric complex Gaussian field[4].

3. Maximal channel capacity

Channel capacity refers to the maximum amount of information that a communication channel can transmit error-free during a given time duration. Several factors influence the channel capacity, including the channel bandwidth, the signal-to-noise ratio (SNR), and the modulation scheme. Shannon's theorem provides a mathematical formula for calculating the maximum channel capacity. Using this theorem, we can analyze the channel capacity of the $N \times N$ channel matrix $\mathbf{H}$

$$C(\mathbf{H}, SNR) = \sum_{i=1}^{N}\log_2\left[1 + \frac{SNR}{N}\sigma_i^2\right] \leq N\log_2\left[1 + \frac{SNR}{N}\sum_{i=1}^{N}(\sigma_i^2/N)\right]\ \mathrm{bits/s/Hz}. \quad \text{(S2)}$$

Equation (S2) relates the channel capacity C with the singular values σ_i of the channel matrix $\mathbf{H}$, where $\sum_{i=1}^{N}\sigma_i^2 = \mathrm{Tr}[\mathbf{H}\mathbf{H}^\dagger]$ can be interpreted as the total power gain of the channel matrix $\mathbf{H}$ if an equal amount of energy is delivered by each source. Then, for the same input power and SNR, the channel capacity is maximized when all singular values are equal, i.e., $\sigma_1 = \sigma_2 = \cdots = \sigma_N$, according to Jensen's inequality.

Identical singular values also correspond to the maximum effective rank, by using the definition of effective rank [Eq. (1) in main text],

$$R_{\text{eff}}(\mathbf{H}) = \exp\left(-\sum_{k=1}^{N} p_k \ln p_k\right) \le \exp\left[N \ln\left(\frac{1}{N}\sum_{k=1}^{N} p_k{}^{-p_k}\right)\right] \le N, \quad \text{(S3)}$$

where $p_k = \sigma_k / (\sum_{i=1}^{N} \sigma_i)$ are the normalized singular values of $\mathbf{H}$. The equal sign holds when $p_k = 1/N$ for all k. Therefore, maximizing the effective rank also maximizes the channel capacity.

4. The roles of the effective rank R_{eff} and parameter w_1 in $\mathcal{G}_1(\mathbf{H})$

In the main text, the first objective function is defined as $\mathcal{G}_1(\mathbf{H}) = [2 - R_{\text{eff}}(\mathbf{H})] + w_1$. Apparently, it has two components, R_{eff} and w_1. The optimization of R_{eff} maximizes the information entropy contained in each channel. And w_1 represents the “degree of diagonalization”, its optimization essentially maximizes the diagonal entries and minimizes the off-diagonal entries, which ensures each channel delivers information from only one source. Both objectives are crucial for optimal channel isolation (OCI). To show this, we compared the experimental results controlled by three different objective functions, $\mathcal{G}_1(\mathbf{H})$ itself, $2 - R_{\text{eff}}(\mathbf{H})$, and w_1, with $\mathbf{H}$ being 2×2. Clearly, the second objective function only targets R_{eff} and ignores the degree of diagonalization, and the third objective function has no explicit consideration of channel capacity. The results are displayed in Supplementary Fig. 5. In Supplementary Fig. 5b, it is seen that the optimization of R_{eff} alone has almost no effect on the degree of diagonalization. This is expected because it is perfectly normal for full-rank matrices to contain non-zero off-diagonal terms. In Supplementary Fig. 5c, we observe that the optimization of w_1, which diminishes the off-diagonal entries, also contributes to an increase in the averaged R_{eff}. However, the variance in R_{eff} is very large among different realizations, which is significantly different from the much smaller red shades shown in Supplementary Fig. 5(b, d), meaning that the channels are still mixed and the channel capacity is not maximized. The reason is that, without the constraint of R_{eff} which enforces the singular values to be almost equal, the diagonal entries can be drastically different in values and some entries may even vanish. Therefore, w_1 alone does not guarantee near orthogonal channels. As a result, OCI demands the simultaneous optimization of R_{eff} and w_1, which is encapsulated in $\mathcal{G}_1(\mathbf{H})$.

5. Ensemble averages of R_{eff} and w_1

The ensemble averages of R_{eff} and w_1 can be obtained numerically and analytically using random matrix theory and probability theory. First, it is straightforward to numerically determine the values. From the discussion in the second section of the Supplementary Information and the Methods section in the main text, the channel matrices are complex Gaussian random matrices (CGRM). Therefore, we generate $10{,}000$ 2×2 CGRM using MATLAB. Statistical analyses are presented as the histograms in Supplementary Fig. 6. The ensemble averages of R_{eff} and w_1 for these random matrices are around 1.72 and 1.18, respectively. These values are indicated by the

dashed lines in Fig. 3c and Fig. 4b in the main text, which are consistent with the experimental measurements.

The ensemble average of R_{eff} can be computed using the probability density function (PDF) of the singular values of CGRM. According to the definition of effective rank [Eq. (1) in main text], R_{eff} of a 2×2 channel matrix is given by

$$R_{\text{eff}} = \exp(E) = \exp\left[-p_1 \ln p_1 - p_2 \ln p_2\right] = \exp\left[-p_1 \ln p_1 - \left(1 - p_1\right) \ln\left(1 - p_1\right)\right], \quad \text{(S4)}$$

where $p_1 = \frac{\sigma_1}{\sigma_1+\sigma_2}$ and $p_2 = \frac{\sigma_2}{\sigma_1+\sigma_2}$ are the normalized singular values, and $p_1 + p_2 = 1$. To simplify the expression in the following, we define a function

$$\alpha(x) \coloneqq \exp[-x \ln x - (1 - x) \ln(1 - x)], \quad \text{(S5)}$$

and rewrite Eq. (S4) as $R_{\text{eff}} = \alpha\left(p_1\right)$. It can be shown that the function $y = \alpha(x)$ monotonically increases in the interval $x \in [0,0.5]$, leading to a one-to-one correspondence between R_{eff} and p_1 in the interval $p_1 \in [0,0.5]$ and the PDF of R_{eff} can be calculated via the PDF of p_1. Consequently, p_1 is obtainable simply as the inverse function $p_1 = \alpha^{-1}(R_{\text{eff}})$. Then, the PDF of R_{eff} is given by[5]

$$\rho_{R_{\text{eff}}}(R_{\text{eff}}) = \rho_1[\alpha^{-1}(R_{\text{eff}})] \left|\frac{\mathrm{d}}{\mathrm{d}R_{\text{eff}}}[\alpha^{-1}(R_{\text{eff}})]\right|, \quad \text{(S6)}$$

where $\rho_1(p_1)$ is the PDF of the normalized singular value p_1. The distribution of singular values of CGRM (the channel matrix $\mathbf{H}$) can be obtained by calculating the eigenvalue distribution of the Wishart-Laguerre ensemble[6] ($\mathbf{HH}^\dagger$ is a Wishart matrix). For 2×2 CGRM with singular values σ_1 and σ_2 (not necessarily ordered), the joint PDF of σ_1 and σ_2 is given by

$$g(\sigma_1, \sigma_2) = \frac{1}{8} \exp\left[-\frac{1}{2}(\sigma_1^2 + \sigma_2^2)\right] (\sigma_1^2 - \sigma_2^2)^2 \sigma_1 \sigma_2. \quad \text{(S7)}$$

The terms $\exp\left[-\frac{1}{2}(\sigma_1^2 + \sigma_2^2)\right]$ and $\sigma_1\sigma_2$ in Eq. (S7) indicate that the singular values are unlikely to take extreme values, and the term $(\sigma_1^2 - \sigma_2^2)^2$ indicates that the two singular values are unlikely to be identical. By using Eq.(S7), we can calculate the PDF of $p_1 = \frac{\sigma_1}{\sigma_1+\sigma_2}$ as

$$\rho_1(p_1) = \int_0^{+\infty} \int_0^{+\infty} g(\sigma_1, \sigma_2) \delta\left(p_1 - \frac{\sigma_1}{\sigma_1+\sigma_2}\right) \mathrm{d}\sigma_1 \mathrm{d}\sigma_2, \quad \text{(S8)}$$

where $\delta(\cdot)$ is the Dirac delta function. By a substitution $r_1 = \frac{\sigma_1}{\sigma_1+\sigma_2}$ and $r_2 = \sigma_2$, we arrive at

$$\rho_1(p_1) = -\frac{6(1-2p_1)^2(-1+p_1)p_1}{[1+2(-1+p_1)p_1]^4}. \quad \text{(S9)}$$

Substituting Eq. (S9) into Eq. (S6), we can obtain the distribution of R_{eff}, which is plotted in Supplementary Fig. 6a as the red solid curve. From this result, the expectation value of R_{eff} for 2×2 CGRM is

$$\bar{R}_{\text{eff}} = \int_1^2 R \cdot \rho_{R_{\text{eff}}}(R) \mathrm{d}R \approx 1.716, \quad \text{(S10)}$$

which conforms with the statistical value and the experimental values [as shown in Fig. 3c and Fig. 4b in the main text].

The ensemble average of the parameter w_1 can be obtained using a similar method. According to the definition of w_1 [Eq. (2) in main text], w_1 of a 2×2 channel matrix is given by

$$w_1 = \frac{|h_{12}|+|h_{21}|}{|h_{11}|+|h_{22}|}, \tag{S11}$$

wherein the magnitudes of the channel matrices entries follow the Rayleigh distribution, and they fall into the range of $(0, +\infty)$. The PDF of w_1, denoted by ρ_{w_1}, can be computed using the sum and quotient rules of random variables. First, we calculate the distributions of the numerator in Eq. (S11), which are given by

$$\begin{aligned} \rho_h(z) &= \int_0^z \rho_R(x)\rho_R(z-x)\mathrm{d}x \\ &= \int_0^z x(z-x)e^{-\frac{1}{2}x^2} e^{-\frac{1}{2}(z-x)^2}\ \mathrm{d}x \\ &= \frac{1}{4}\,\mathrm{e}^{-\frac{1}{2}z^2}\left[2z + \sqrt{\pi}e^{\frac{1}{4}z^2}(z^2-2)\mathrm{Erf}\left(\frac{z}{2}\right)\right] \end{aligned}, \tag{S12}$$

where $z = |h_{12}| + |h_{21}|$, $\rho_R(x) = x\mathrm{e}^{-\frac{1}{2}x^2}$ is the PDF of the Rayleigh distribution, and $\mathrm{Erf}(x) = \frac{2}{\sqrt{\pi}}\int_0^x e^{-t^2}\mathrm{d}t$ is the error function with t being variable to be integrated over. The PDF of the denominator in Eq. (S11) apparently has the same form. The distribution of w_1 then follows as

$$\rho_{w_1}(w_1) = \int_0^{+\infty} z\rho_h(z)\rho_h(w_1 z)\mathrm{d}z. \tag{S13}$$

The distribution $\rho_{w_1}(w_1)$ is plotted in Supplementary Fig. 6b as the red solid curve, which agrees well with the numerical result. The expectation value of the parameter w_1 can be determined as follows:

$$\bar{w}_1 = \int_0^{+\infty} w_1\rho_{w_1}(w_1)\mathrm{d}w_1 \approx 1.184. \tag{S14}$$

This result agrees well with the experimental values shown in Fig. 3c and Fig. 4b in the main text.

6. Measurement of R_{eff} and w_1 in the frequency range of 500-4000 Hz

Here, we provide experimental evidence that optimizing the channel isolation metric at a single frequency does not affect the channel isolation metric at frequencies outside the coherence bandwidth (about ±4.2 Hz) from a statistical averaging perspective. We performed the same experiment as shown in Fig. 3 in the main text, where we optimized the objective function $\mathcal{G}_1(\mathbf{H})$ [Eq. (3) in the main text] at 1300 Hz but provided measurements of the effective rank R_{eff} and diagonalization degree w_1 over a wider frequency range of 500-4000 Hz.

The results are shown in Supplementary Fig. 7. It is seen that only at 1300 Hz R_{eff} and w_1 do approach the desired values of 2 and 0, respectively. However, at frequencies outside the coherence bandwidth, they are at the values derived from the Rayleigh channel characteristics, approaching values of 1.7 and 1.2, respectively.

7. Objective functions for optimizing 6×2 and 4×2 channel matrices

Here, we present the objective functions used for achieving the effects shown in Fig. 6 in the main text. For the scenario described by 6×2 channel matrices [Fig. 6(a, b, c)], the entries to preserve are denoted by $A_1 = \{|h_{11}|, |h_{21}|, |h_{31}|, |h_{42}|, |h_{52}|, |h_{62}|\}$, and the entries to eliminate are denoted by $A_0 = \{|h_{12}|, |h_{22}|, |h_{32}|, |h_{41}|, |h_{51}|, |h_{61}|\}$. The objective function is

$$\mathcal{G}_4(\mathbf{H}) = |2 - R_{\text{eff}}(\mathbf{H})| + \frac{\text{sum}(A_0)}{\text{sum}(A_1)} + \frac{\text{std}(A_1)}{\text{max}(A_1)}, \tag{S15}$$

where $\text{sum}(\cdot)$, $\text{std}(\cdot)$ and $\max(\cdot)$ represent the summation, standard deviation, and maximum value, respectively. The minimization of the objective function $\mathcal{G}_4$ achieves three objectives: (i) the first term drives the effective rank R_{eff} to 2, (ii) the second term serves to minimize the entries in A_0, and (iii) the third term ensures the uniformity of the entries in A_1.

Similarly, for the scenario illustrated in Fig. 6(d, e, f), which are described by 4×2 channel matrices, the goal is to preserve $A_1 = \{|h_{11}|, |h_{21}|, |h_{22}|, |h_{32}|\}$ and eliminate $A_0 = \{|h_{12}|, |h_{31}|, |h_{41}|, |h_{42}|\}$. Thus, the objective function is designed as

$$\mathcal{G}_5(\mathbf{H}) = |1.9286 - R_{\text{eff}}(\mathbf{H})| + \frac{\text{sum}(A_0)}{\text{sum}(A_1)} + \frac{\text{std}(A_1)}{\text{max}(A_1)}. \tag{S16}$$

Note that because we require the entire row 4 to vanish, the upper bound of the effective rank of the 4×2 channel matrix is ~1.9286, instead of 2.

8. OCI over a continuous band of frequency

The acoustic reconfigurable metasurfaces (ARMs) are capable of modulating the phase of the reflected wave over a broad bandwidth, which gives rise to the possibility of controlling acoustic channels over a finite band of frequency. In order to verify this capability, we have performed 30 independent experiments to demonstrate the optimization of 2×2 channel matrices (based on the minimization of $\mathcal{G}_1$) over 1350 Hz to 1450 Hz. The R_{eff} is increased to approximately 1.883 within the 100 Hz bandwidth, and the w_1 is reduced to approximately 0.400 (corresponding to a decrease of about 8 dB), as shown in Supplementary Fig. 8.

To enable OCI to be performed across a continuous frequency band, we defined the objective function as follows:

$$\mathcal{G}_6 = (2 - \bar{R}_{\text{eff}}) + \bar{w}_1 + \text{std}(R_{\text{eff}}) + \text{std}(w_1), \tag{S17}$$

where $\bar{R}_{\text{eff}} = \frac{1}{N}\sum_{n=0}^{N} R_{\text{eff}}[\mathbf{H}(f_0 + n \times \delta f)]$, $\bar{w}_1 = \frac{1}{N}\sum_{n=0}^{N} w_1[\mathbf{H}(f_0 + n \times \delta f)]$ are the spectrally averaged effective rank and degree of diagonalization, and $\text{std}(\cdot)$ represent their standard deviations in the spectrum. We measured the channel matrix at 26 frequencies in the range of 1350 and 1450 Hz with a frequency resolution of 4 Hz, i.e. $N = 26, f_0 = 1350\ \text{Hz}, \delta f = 4\ \text{Hz}$. The definitions of R_{eff} and w_1 follow Eqs. (1) and (2) in the main text.

9. The optimization algorithm

We utilized a climbing algorithm to minimize the objective function, as illustrated in Supplementary Fig. 9. This algorithm does not require prior knowledge of the acoustic field, but only relies on configuring the states of ARMs. The ARMs consist of 200 units that can only switch between Open state and Closed state, denoted as “0” and “1”. The iterative process is as follows:

(1) Set all unit states to “0” as the *first initial state* and measure the channel matrix in the room. The value of the objective function is then computed.

(2) Randomly select *M* units (*M* being a randomly selected integer between 1 and 15) and switch their states based on the initial state (changing “0” to “1” and vice versa) to create a new state. Then measure the channel matrix in the room and calculate the objective function.

(3) Compare the objective functions of the initial and new states, then select the better state as the *new initial state*. If the objective function remains unchanged or increases, revert to the *original initial state*.

(4) Repeat steps (2) to (3) until the objective function converges to the desired value.

Supplementary References

1. Yon, S., Tanter, M. & Fink, M. Sound focusing in rooms: The time-reversal approach. *J. Acoust. Soc. Am.* **113**, 1533–1543 (2003).
2. Ma, G. *et al.* Towards anti-causal Green's function for three-dimensional sub-diffraction focusing. *Nat. Phys.* **14**, 608–612 (2018).
3. Nélisse, H. & Nicolas, J. Characterization of a diffuse field in a reverberant room. *J. Acoust. Soc. Am.* **101**, 3517–3524 (1997).
4. Mason, W. P. & Thurston, R. N. *Physical acoustics: principles and methods*. vol. 17 (Acad. Press, 1984).
5. Dekking, M., Kraaikamp, C., Lopuhaä, H. P. & Meester, L. E. *A modern introduction to probability and statistics: understanding why and how*. (Springer, 2005).
6. Shen, J. On the singular values of gaussian random matrices. *Linear Algebra Its Appl.* **326**, 1–14 (2001).

Supplementary Figures

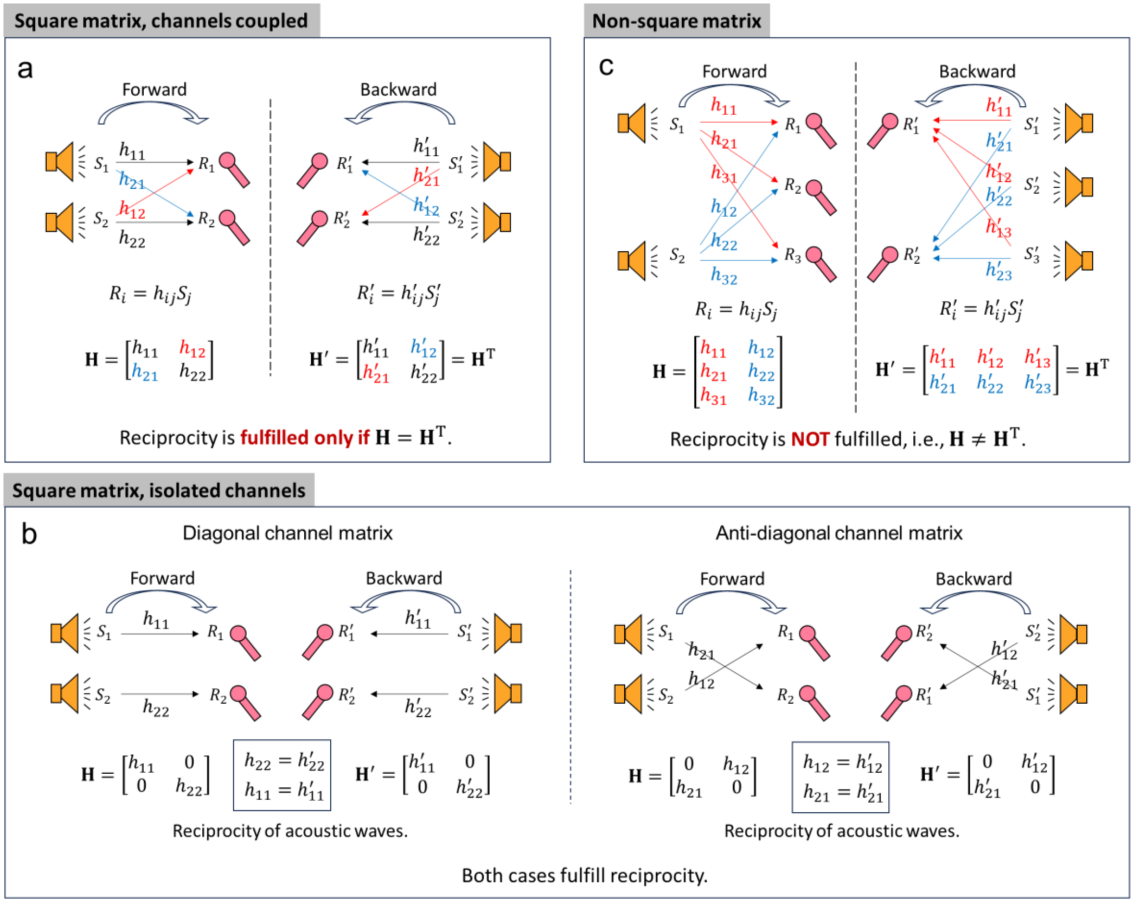

Supplementary Fig. 1 On the reciprocity of channel matrices. **a** The reciprocity condition requires the channel matrix to be transpose-invariant. **b** Reciprocity is satisfied when the optimal channel isolation is obtained. **c** Reciprocity is not satisfied if the channel matrix is not square.

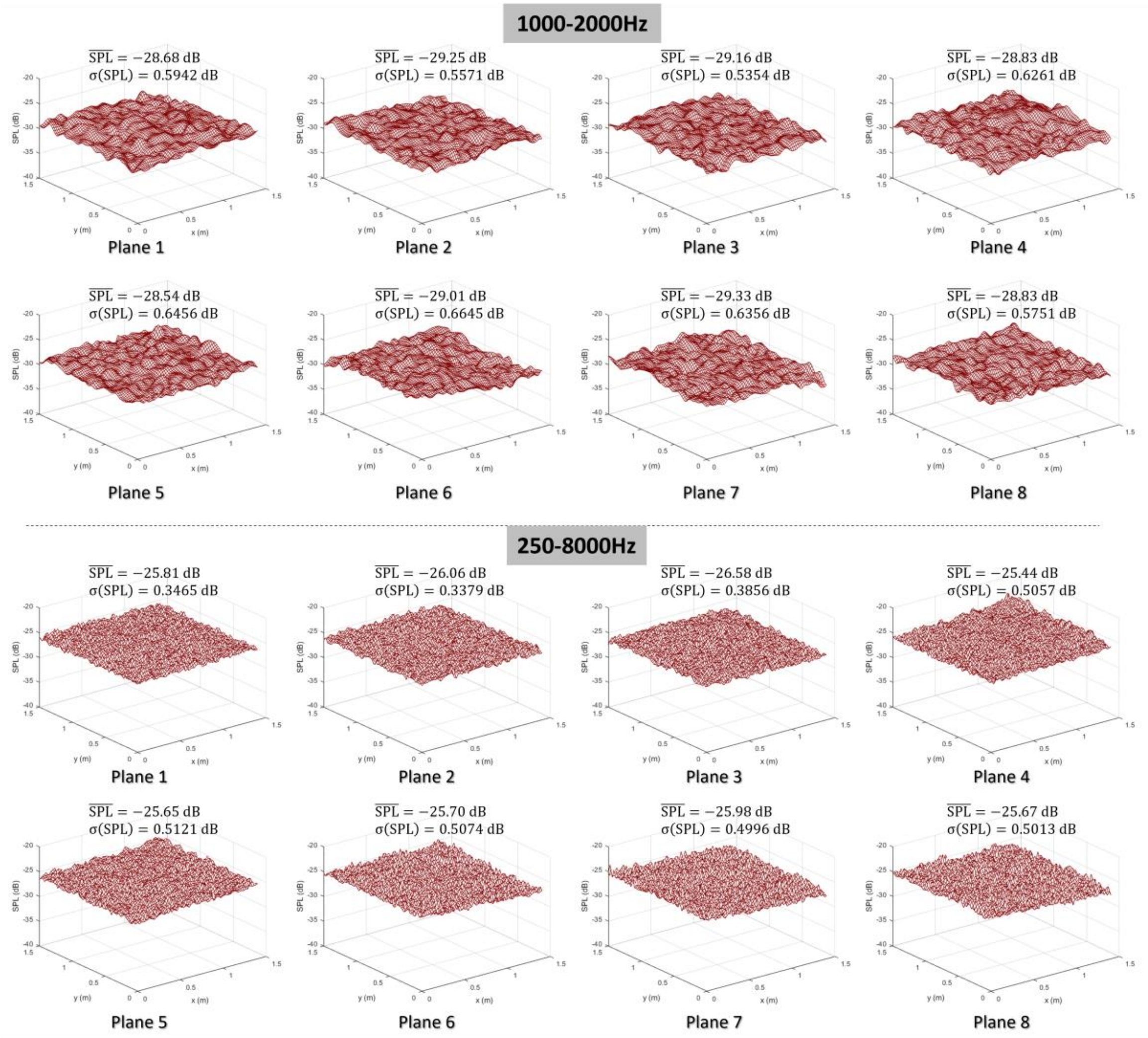

Supplementary Fig. 2 The spatial distributions of the spectral averages of the sound pressure levels (SPL) are illustrated for Planes 1-8. The calculations for the SPL are conducted for two frequency ranges: 1000-2000 Hz (upper) and 250-8000 Hz (bottom). The spatial means ($\overline{SPL}$) and standard deviations [$\sigma(SPL)$] of SPL are marked above each panel. Both frequency ranges exhibit a standard deviation of less than 0.7 dB.

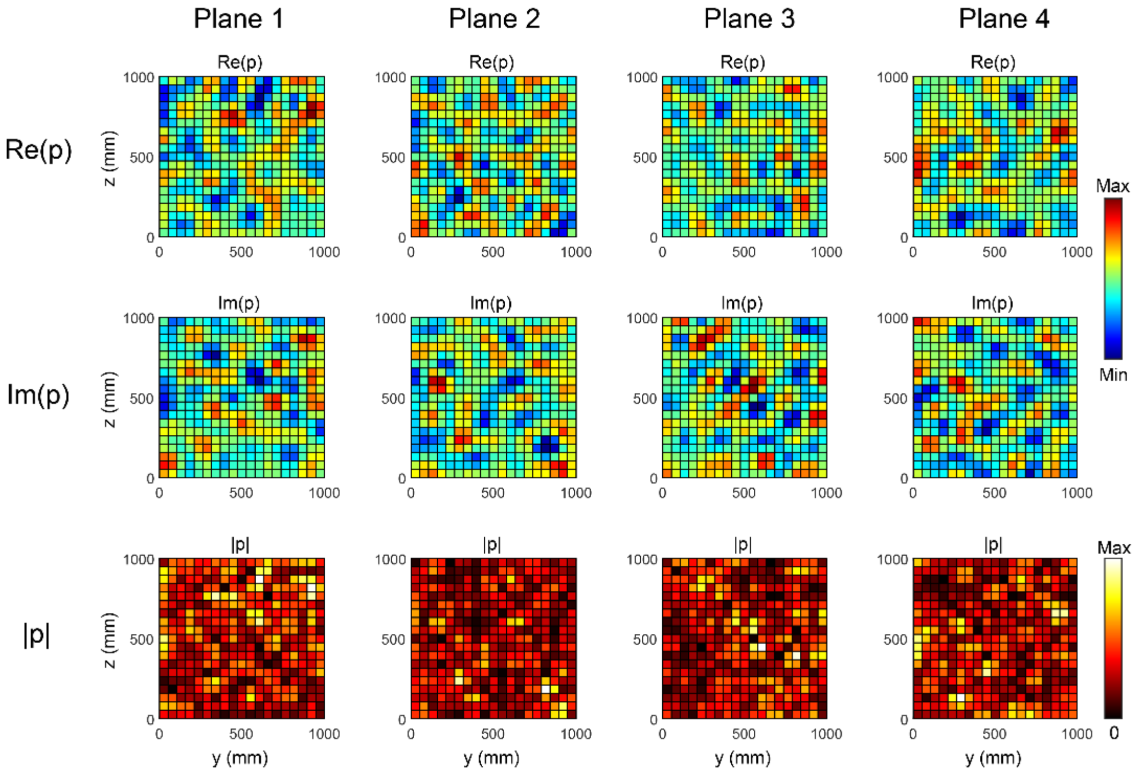

Supplementary Fig. 3 Two-dimensional scans of the sound fields at different positions in the room at 1300 Hz. Each column is one set of experiment.

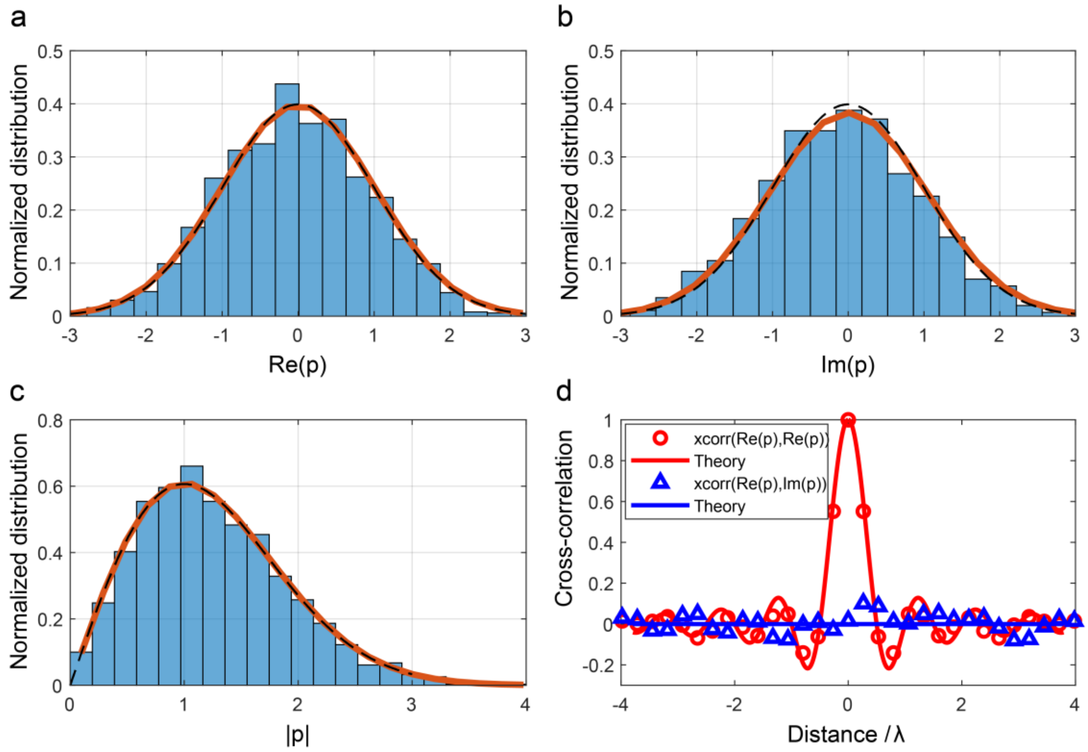

Supplementary Fig. 4 The statistical distributions of (**a**) $\mathrm{Re}(p)$, (**b**) $\mathrm{Im}(p)$ and (**c**) $|p|$. The red solid curves are numerical fitting from the experimental data, and the black dashed curves represent theoretical models: Gaussian distributions in (**a**, **b**) and Rayleigh distribution in (**c**). The values of the sound pressure are normalized by their standard deviation. **d** The spatial correlations of the sound fields.

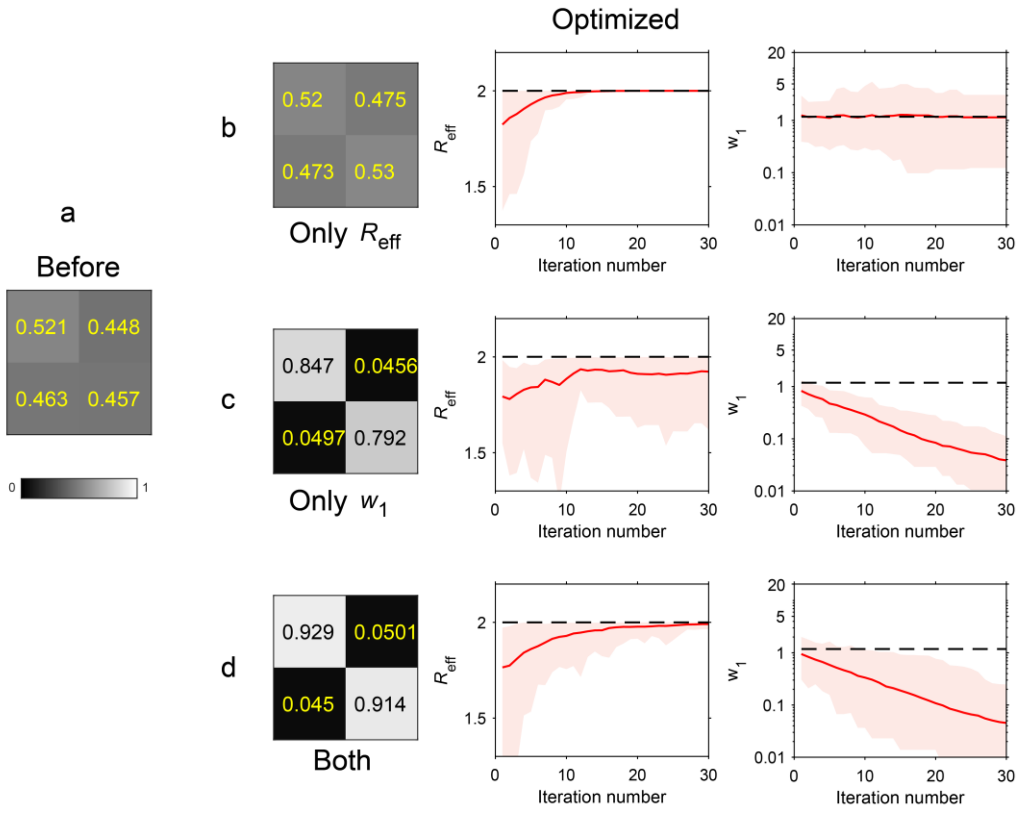

Supplementary Fig. 5 The roles of R_{eff} and w_1 in objective function $\mathcal{G}_1(\mathbf{H})$. **a** The magnitude-averaged entries of the channel matrix before the optimization. (**b, c, d)** correspond to the results obtained using $2 - R_{\text{eff}}(\mathbf{H})$, w_1, and $\mathcal{G}_1(\mathbf{H}) = 2 - R_{\text{eff}}(\mathbf{H}) + w_1$ as the objective function, respectively. **b** Only the effective rank is optimized, and R_{eff} is increased to the upper bound of 2 (middle), but w_1 experiences no improvement (right). **c** Only w_1 is minimized, and w_1 is significantly suppressed to about 0.03 (right). R_{eff} is also improved to 1.92 (middle) due to the suppression of the off-diagonal entries (left). However, it is far from the ideal value of 2. **d** R_{eff} and w_1 are optimized at the same time. The R_{eff} is increased to 1.99 (middle) and w_1 is reduced to about 0.04 (right). In (**b, c, d)** the red curves are the averaged values and the red shades depict the ranges of the respective values in all realizations. The measured frequency is 1300 Hz. The black dashed lines mark $R_{\text{eff}} = 2$ in the middle column and the black dashed lines in the right column indicate the expected value of w_1 in the uncontrolled case, i.e., $w_1 \approx 1.2$.

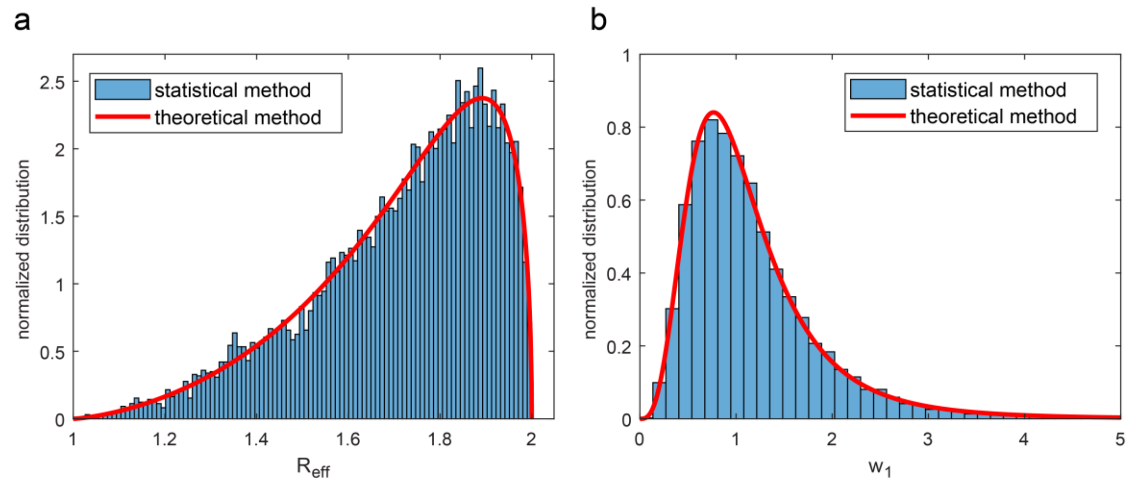

Supplementary Fig. 6 The numerically obtained distributions of R_{eff} (a) and w_1 (b) are depicted as histograms. The red solid curves are the theoretical results of random matrices and probability theory.

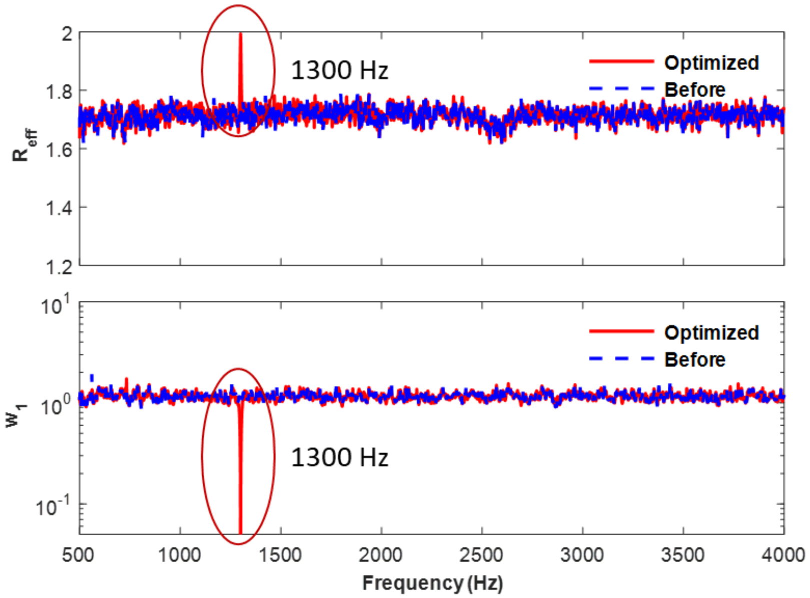

Supplementary Fig. 7 Measurement of R_{eff} and w_1 in the frequency range of 500-4000 Hz, with optimization performed solely at 1300 Hz.

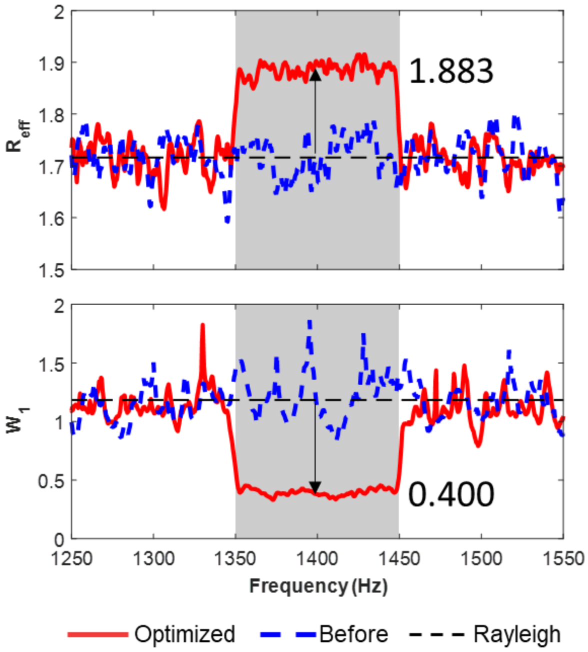

Supplementary Fig. 8 Optimization of 2×2 channel matrices by minimizing $\mathcal{G}_1(\mathbf{H})$ over a continuous band of frequency spanning 100 Hz.

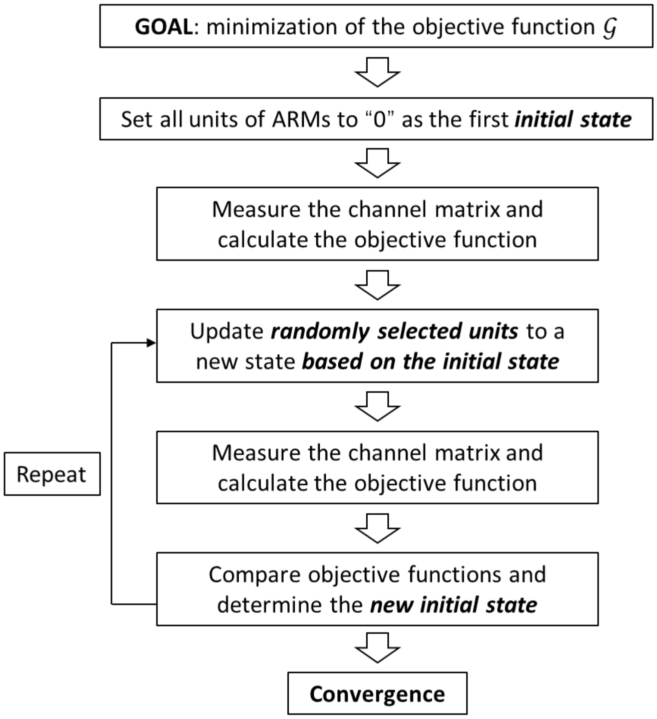

Supplementary Fig. 9 Optimization procedure of the climbing algorithm.
