# Supplementary material for: Optimizing multi-user indoor sound communications with acoustic reconfigurable metasurfaces
Source: Nat Commun. 2024 Feb 10;15:1270. doi: 10.1038/s41467-024-45435-4 (PMC10858938; doi:10.1038/s41467-024-45435-4)
Supplement: Supplementary file 3 — Description of Additional Supplementary Files [file 41467_2024_45435_MOESM3_ESM.pdf]

Description of Additional Supplementary Files:

Supplementary Movie 1: Demonstration of optimal channel isolation with simultaneous music playback.
